# MSANet: a multi-scale spatiotemporal adaptive network for EEG-based driver fatigue recognition

**DOI:** 10.3389/fnins.2026.1939908

**Published:** 2026-10-06

**Authors:** Peipei Gu, Ruibo Wang, Ming Zhang, Naian Xiao, Fangqin Liu, Bin Jiang, Jiayang Guo, Xiaosong Peng

**Affiliations:** 1 School of Software Engineering, Zhengzhou University of Light Industry, Zhengzhou, China; 2 Department of Pediatrics, Zhongshan Hospital, Xiamen University, Xiamen, Fujian, China; 3 Department of Neurology, The Third Hospital of Xiamen, Xiamen, Fujian, China; 4 Department of Neurology and Geriatrics, Fujian Institute of Geriatrics, Fujian Medical University Union Hospital, Fuzhou, Fujian, China; 5 Xiamen Shuimu Ruizhi Intelligent Technology Co., Ltd., Xiamen, China; 6 Department of Neurology and Department of Neuroscience, The First Affiliated Hospital, School of Medicine, Xiamen University, Xiamen, China; 7 Xiamen Medical Quality Control Center for Neurology, Xiamen, China; 8 Department of Geriatrics, The Third Hospital of Xiamen, Xiamen, Fujian, China; 9 Department of Geriatrics, The First Affiliated Hospital of Xiamen University, Xiamen, Fujian, China

**Keywords:** adaptive classification, cross-subject transfer learning, driver fatigue recognition, dynamic graph learning, electroencephalography, multi-scale temporal modeling

## Abstract

Electroencephalography (EEG)-based driver fatigue recognition remains challenging because fatigue-related patterns are distributed across multiple temporal scales, inter-channel relationships vary across samples, and inter-individual differences complicate cross-subject transfer. To address these issues, a multi-scale spatiotemporal adaptive network (MSANet) is proposed for EEG-based driver fatigue recognition. The framework introduces a multi-granularity patch temporal gate (MGPTG) to model locally continuous temporal contexts, stable graph node enhancement (SGNE) to enhance channel-node features before dynamic graph construction, and an adaptive classifier gate (ACG) to combine cosine-based and affine decision geometries. MSANet was evaluated on two public simulated-driving EEG datasets using a cross-subject transfer protocol, in which the target-subject samples were stratified with a partition ratio of 0.5 into a labeled adaptation subset and a held-out test subset. MSANet achieved an accuracy (ACC) of 83.64% and an F1-score (F1) of 83.74% on Dataset I, and 74.64% ACC and 72.92% F1 on Dataset II. The results indicate strong numerical performance on Dataset I and competitive performance on Dataset II. Subject-level statistical analysis was additionally conducted to distinguish mean performance differences from differences that are consistently supported across subjects. Ablation results showed lower mean performance after removing MGPTG, SGNE, or ACG under the evaluated settings. These findings support further evaluation of the proposed stage-specific temporal, graph-node, and classification mechanisms for cross-subject transfer in EEG-based driver fatigue recognition.

## 1 Introduction

Driver fatigue impairs vigilance, reaction speed, and decision-making ability, increasing the risk of driving errors and traffic accidents. Existing driver-monitoring approaches mainly rely on vehicle behavior, visual cues, or physiological signals. Vehicle- and vision-based indicators are convenient but can be affected by road conditions, illumination, occlusion, and individual driving styles. In contrast, electroencephalography (EEG) provides a direct measure of neural activity associated with arousal and attention and may reveal fatigue-related changes before overt driving-performance degradation becomes apparent (Cao et al., 2019; Stancin et al., 2021). Reliable driver-state monitoring may be particularly relevant to older drivers because age-associated changes in attention, cognition, and driving behavior can increase safety concerns (Menze et al., 2025; Hettiarachchige et al., 2025). However, the present study evaluates a general EEG-based fatigue-recognition framework using public datasets that were not designed for age-specific analysis. Accordingly, the relevance to older drivers is considered a potential application context rather than an empirically validated age-specific conclusion.

Traditional EEG-based fatigue recognition has relied on spectral power, entropy, wavelet coefficients, and manually constructed connectivity features (Min et al., 2021; Yang et al., 2025; Subasi et al., 2022). Although effective in selected settings, such pipelines depend strongly on predefined feature representations and classifier design. Recent deep-learning approaches have therefore shifted toward multidimensional representation learning, including dynamic graph modeling (Wang et al., 2021), temporal–channel attention (Chen et al., 2023), temporal–spatial–spectral fusion (Gao et al., 2024a,b), and brain-region-aware graph learning (Hu et al., 2024). More recent studies have further explored mutual-information-based graph attention (Chen et al., 2026a), entropy-informed residual learning (Chen et al., 2026b), and adaptive dynamic convolution across spectral, spatial, and temporal domains (Zhou et al., 2026). A recent review summarizes this transition across public datasets, preprocessing, feature extraction, evaluation metrics, and deep-learning architectures (Chen et al., 2026d), while multi-band feature optimization remains a complementary direction for characterizing fatigue-related neural signatures (Chen et al., 2026c).

Despite these advances, cross-subject transfer remains challenging because EEG distributions vary substantially across individuals. Cross-subject convolutional and residual-shrinkage models have been developed to reduce sensitivity to inter-subject variability (Cui et al., 2023; Feng et al., 2025). Dynamic-graph and unseen-driver frameworks have further investigated subject-level generalization under more stringent evaluation settings (Xu et al., 2025; Chen et al., 2026e). In particular, NHGNet provides a strong dynamic-graph formulation for EEG-based driver fatigue recognition. However, dynamic graph construction alone does not address all modeling requirements of the temporal–graph pipeline. In particular, challenges remain in locally continuous temporal modeling, pre-adjacency node representation, and the final decision geometry.

Three issues are particularly relevant in this setting. First, conventional multi-scale temporal modeling does not explicitly capture locally continuous evidence across neighboring intervals. Second, dynamic adjacency estimation may be affected when compact channel-node representations are used directly for graph construction without an explicit node-enhancement stage. Third, a single classifier geometry may be insufficient when target-subject adaptation alters both the magnitude and orientation of learned representations.

Accordingly, MSANet introduces three dedicated components within a temporal–graph backbone, as illustrated in Figure 1. A multi-granularity patch temporal gate (MGPTG) employs overlapping multi-granularity patch gating to model locally continuous temporal contexts before temporal compression. Stable graph node enhancement (SGNE) introduces bounded residual node enhancement immediately before dynamic adjacency estimation, so that graph construction is performed on enhanced channel-node representations. An adaptive classifier gate (ACG) introduces gated dual-geometry classification by combining normalized cosine and affine decision spaces after graph convolution. These components operate at distinct stages of the processing pipeline and address different modeling objectives beyond dynamic graph construction alone.

**Figure 1:**
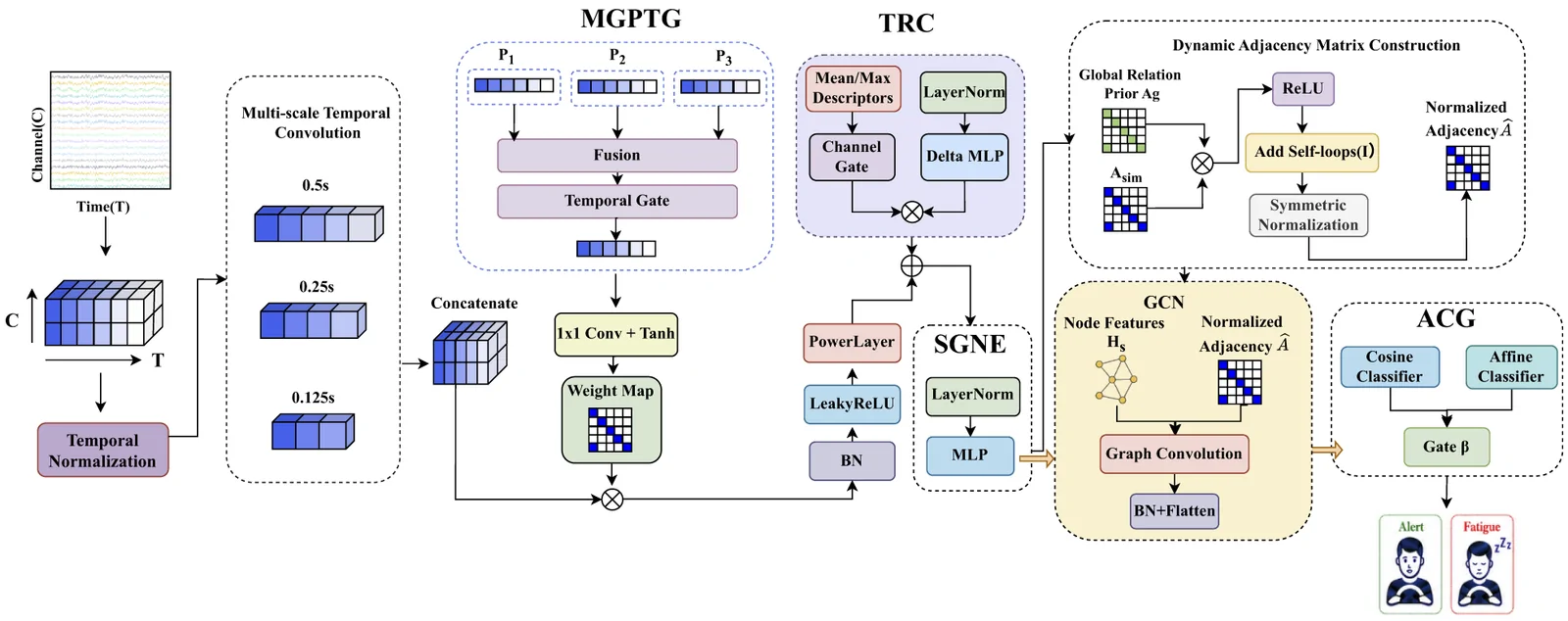
Overall architecture of MSANet.

The main contributions of this work are summarized as follows:

- A temporal–graph framework is developed for cross-subject transfer in EEG-based driver fatigue recognition, integrating dedicated mechanisms at the temporal modeling, pre-adjacency node representation, and classification stages.
- MGPTG introduces overlapping multi-granularity patch gating for locally continuous temporal modeling, while SGNE introduces bounded residual node enhancement immediately before dynamic adjacency estimation.
- ACG introduces gated dual-geometry classification by adaptively combining cosine-based and affine decision spaces. The complete framework is evaluated on two public simulated-driving EEG datasets under a common cross-subject transfer protocol.

The remainder of this paper is organized as follows. Section 2 describes the proposed methodology, Section 3 presents the experimental settings, Sections 4, 5 report and discuss the results, respectively, and Section 6 concludes the study.

## 2 Methodology

### 2.1 Overall architecture

Let an EEG segment be denoted by **X**∈ℝ^*B*×*C*×*T*^, where *B*, *C*, and *T* represent the batch size, number of EEG channels, and number of temporal samples, respectively. Each channel is standardized along the temporal dimension before entering the network.

As illustrated from left to right in Figure 1, the normalized EEG segment is first processed by three temporal convolution branches. The concatenated multi-scale representation is then modulated by MGPTG through bounded residual temporal gating. A separate temporal weighting pathway is subsequently applied, followed by energy-based pooling. Temporal residual calibration (TRC) further recalibrates the resulting compact channel-wise features. SGNE then enhances these node representations before the dynamic adjacency matrix is estimated. A graph convolutional network (GCN) layer produces the final graph representation, which is classified by ACG. The calibration and enhancement pathways also use batch normalization (BN) and a multilayer perceptron (MLP), whose roles are detailed in the following subsections.

The architecture therefore follows three consecutive stages: temporal encoding, graph learning, and classification. MGPTG, SGNE, and ACG are the three proposed stage-specific components and correspond directly to the three methodological limitations identified in the Introduction: local temporal continuity, node enhancement before graph estimation, and complementary decision geometries. The multi-scale temporal encoder, temporal weighting and pooling, TRC, dynamic adjacency construction, and GCN constitute the temporal–graph backbone.

### 2.2 MGPTG-based multi-scale temporal learning

To capture fatigue-related EEG dynamics over complementary temporal ranges, the temporal encoder first applies three parallel channel-wise depthwise convolution branches with kernel lengths of 0.5*f*_*s*_, 0.25*f*_*s*_, and 0.125*f*_*s*_, where *f*_*s*_ denotes the sampling frequency. At the common sampling rate of 128 Hz, these settings correspond to kernel sizes of 64, 32, and 16 samples, respectively. Each branch contains 30 depthwise filters corresponding to the 30 retained EEG channels, allowing temporal patterns to be extracted independently within individual channels without introducing premature inter-channel mixing. The three branches therefore provide complementary temporal receptive fields for the same EEG segment. Their outputs are subsequently concatenated along the scale dimension to form the multi-scale representation **Z**∈ℝ^*B*×*C*×*S*×*T*^′, where *S* = 3 denotes the number of temporal scales. This channel-wise design preserves electrode-specific temporal characteristics at the encoding stage, while inter-channel dependencies are modeled subsequently during graph learning.

Building on the multi-scale representation **Z**, MGPTG further models temporal salience over overlapping local intervals before subsequent feature weighting and temporal compression operations. As illustrated in Figure 2, **Z** is first pooled across the channel and scale dimensions to obtain a global temporal descriptor. Three parallel patch branches are then used to capture local temporal contexts at different granularities. Because the two datasets contain EEG segments with different temporal durations, dataset-specific patch configurations are adopted. Patch lengths of 16, 24, and 32 samples are used for Dataset I, whereas 8, 16, and 24 samples are used for Dataset II. The stride of each branch is set to half of the corresponding patch length, thereby preserving overlap between adjacent temporal intervals. Each branch consists of a temporal patch convolution, a LeakyReLU activation, and a 1 × 1 projection. The branch outputs are resized to the common temporal length *T*′, concatenated along the branch dimension, and subsequently fused through a 1 × 1 convolution followed by a tanh activation to generate the temporal gate **G**.

**Figure 2:**
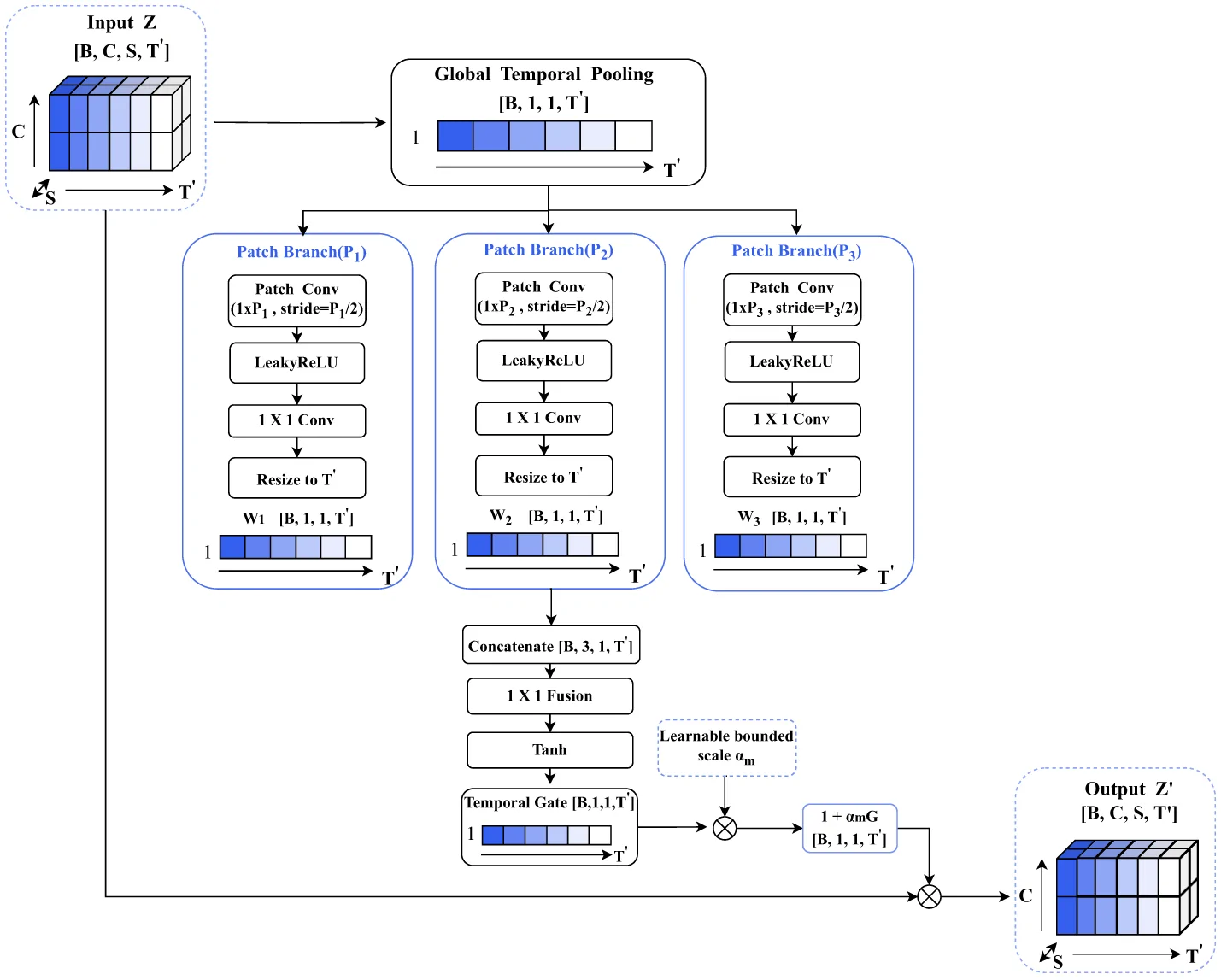
Architecture of MGPTG. The multi-scale representation **Z** is processed by three overlapping patch branches to generate the temporal gate **G**, which modulates the original representation through bounded residual scaling.

The generated temporal gate **G** is applied to the original multi-scale representation through bounded residual modulation:


$$\begin{array}{l} Z^{\prime }=Z⊙\left(1+\alpha_{m}G\right),\;\alpha_{m}=\alpha_{m}^{\max}\sigma \left(a_{m}\right), \end{array}$$ (1)

where *a*_*m*_ is a learnable scalar, $\alpha_{m}^{\max}$ bounds the residual scale, and σ(·) denotes the sigmoid function. The bounded residual formulation preserves the original multi-scale representation while allowing the patch-derived gate to emphasize locally salient temporal regions without replacing the underlying features.

Following MGPTG modulation, a separate 1 × 1 convolution followed by a tanh activation generates the feature-weight map used in the subsequent temporal weighting pathway, as shown in Figure 1. BN and LeakyReLU activation are then applied. A power layer computes log-energy over sliding temporal intervals, after which the scale and pooled-time dimensions are flattened into compact channel-wise features. TRC subsequently recalibrates these features using complementary mean and maximum descriptors. The descriptor-based channel gate is combined with a residual calibration pathway composed of layer normalization and an MLP before the enhanced features are passed to SGNE.

### 2.3 SGNE-based dynamic graph learning

Since the dynamic adjacency matrix is estimated from channel-node representations, the quality of node features directly affects the reliability of graph construction. Therefore, SGNE is applied before adjacency estimation to enhance the channel-node representations while preserving the original feature structure. As shown in Figure 3, SGNE consists of layer normalization, a shared MLP, and a bounded residual pathway.

**Figure 3:**
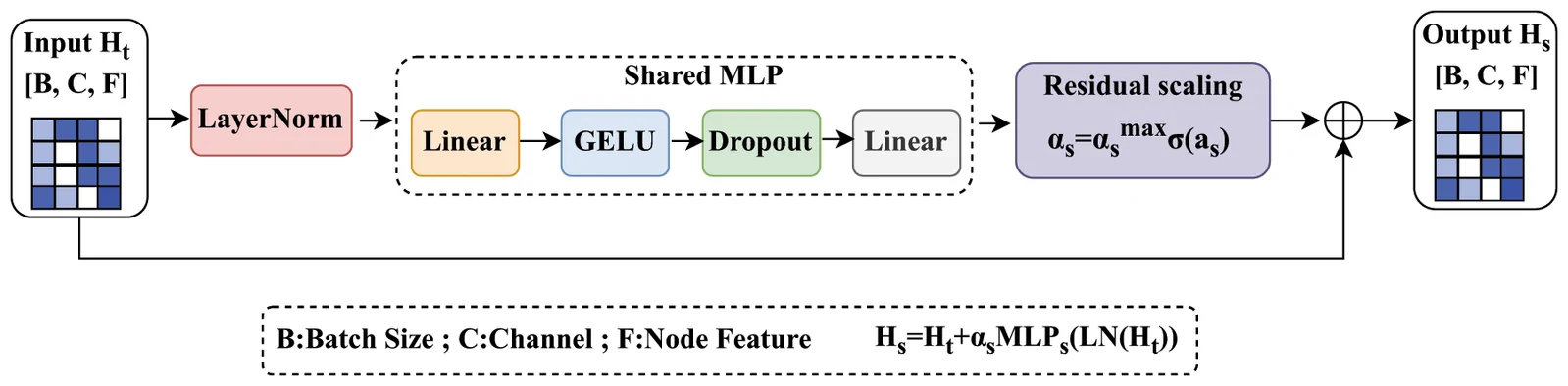
Architecture of SGNE. The module enhances channel-node representations through a shared MLP and a bounded residual pathway before dynamic adjacency estimation.

**Figure 4:**
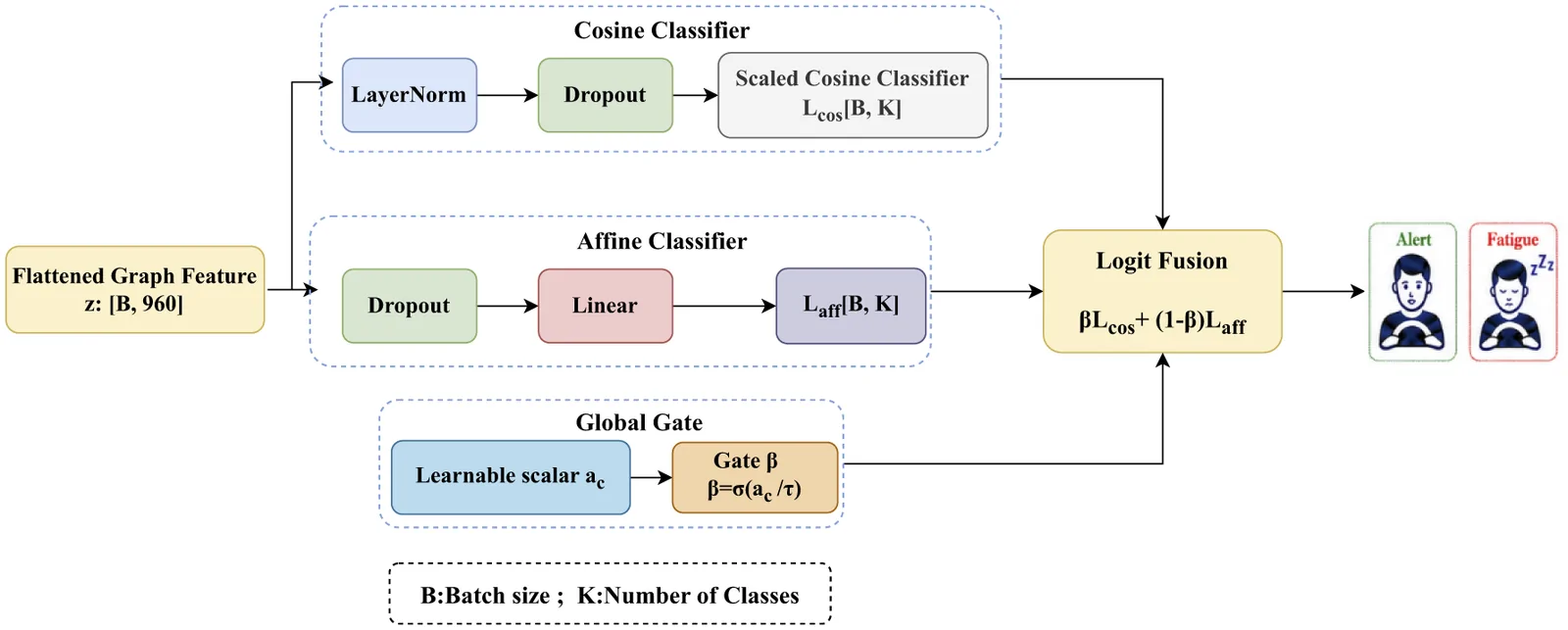
Architecture of ACG. The module combines cosine-based and affine decision geometries through a learnable global gate for dual-geometry classification.

The enhanced node representation is formulated as


$$\begin{array}{l} H_{s}=H_{t}+\alpha_{s}{\mathrm{MLP}}_{s}\left(\mathrm{LayerNorm}\left(H_{t}\right)\right),\;\alpha_{s}=\alpha_{s}^{\max}\sigma \left(a_{s}\right), \end{array}$$ (2)

where **H**_*t*_ denotes the TRC output, *a*_*s*_ is a learnable scalar, $\alpha_{s}^{\max}$ controls the maximum residual scale, and σ(·) denotes the sigmoid function. The MLP adopts a shared Linear–GELU–Dropout–Linear structure across EEG channel nodes. The corresponding dimensions are set to 963 → 1, 926 → 963 for Dataset I and 195 → 390 → 195 for Dataset II, with a dropout rate of 0.1.

The enhanced node representation **H**_*s*_ is then used for dynamic adjacency construction. A scaled dot-product similarity matrix is first computed as


$$\begin{array}{l} A_{sim}=\frac{H_{s}H_{s}^{⊤}}{\sqrt{F}}, \end{array}$$ (3)

where *F* denotes the node feature dimension. To incorporate a shared global relation prior, a trainable relation matrix is symmetrized before use, yielding **A**_*g*_, which is combined element-wise with the sample-specific similarity matrix:


$$\begin{array}{l} A=A_{sim}⊙A_{g}. \end{array}$$ (4)

Negative affinities are removed through rectification, after which self-loops are added:


$$\begin{array}{l} \tilde{A}=\mathrm{ReLU}\left(A\right)+I. \end{array}$$ (5)

The adjacency matrix is then symmetrically normalized as


$$\begin{array}{l} \hat{A}=D^{-\frac{1}{2}}\tilde{A}D^{-\frac{1}{2}}, \end{array}$$ (6)

where **D** is the degree matrix of $\tilde{A}$.

A single GCN layer is subsequently applied using the normalized adjacency matrix $\hat{A}$ to propagate information among EEG channel nodes. The GCN maps the node features to 32 dimensions, resulting in a *C*×32 node representation. Specifically, the feature mappings are 963 → 32 for Dataset I and 195 → 32 for Dataset II. The resulting graph representation is flattened into a 960-dimensional feature vector and delivered to ACG.

### 2.4 ACG-based dual-geometry classification

After the GCN layer, the node representations are flattened into a graph-level feature vector **z**∈ℝ^*B*×960^ for final classification. Since target-subject adaptation may introduce variations in both feature magnitude and direction, a single decision geometry may limit the flexibility of the classifier. Therefore, ACG combines complementary cosine-based and affine decision geometries at the classification stage.

As illustrated in Figure 4, ACG contains two parallel classifier branches. The cosine branch uses normalized feature representations and scaled cosine similarity to emphasize the angular relationship between the graph-level feature and class prototypes, whereas the affine branch retains magnitude- and bias-related information. The two branches produce logit matrices $L_{\text{cos}}\in {\mathbb{R}}^{B\times K}$ and $L_{\text{aff}}\in {\mathbb{R}}^{B\times K}$, respectively. Their outputs are fused through a learnable global gate as


$$\begin{array}{l} L=\beta L_{\text{cos}}+\left(1-\beta \right)L_{\text{aff}}, \end{array}$$ (7)

where **L**∈ℝ^*B*×*K*^ denotes the fused logits and the fusion coefficient β is determined by


$$\begin{array}{l} \beta =\sigma \left(a_{c}/\tau \right), \end{array}$$ (8)

where *a*_*c*_ is a learnable scalar, σ(·) denotes the sigmoid function, and τ controls the gate temperature. The temperature is set to 1.5 for Dataset I and 2.0 for Dataset II.

By combining magnitude-invariant cosine geometry and magnitude-sensitive affine geometry, ACG provides a learnable dual-geometry classification formulation for cross-subject EEG recognition.

## 3 Experiments

### 3.1 Datasets

Two public EEG-based driver fatigue datasets were used to evaluate the proposed framework under different segment lengths and labeling protocols. For a consistent model input, 30 common EEG channels were retained in both datasets, and all EEG signals were represented at a sampling rate of 128 Hz.

#### 3.1.1 Dataset I

Dataset I is derived from the sustained-attention driving dataset released by Cao et al. (2019). The processed cohort used in this study contains 11 subjects retained for analysis, each with available alert and fatigue samples. Each trial is represented as 1 × 30 × 384, corresponding to a 3-s EEG segment with 30 channels and 384 temporal samples. Alert and fatigue labels follow the reaction-time-based criterion adopted in the original dataset and the comparison framework, with samples selected from clearly separated vigilance levels. Dataset I therefore provides the longer-window setting for evaluating cross-subject transfer performance.

#### 3.1.2 Dataset II

Dataset II is based on the simulated-driving EEG data described by Min et al. (2017) and contains 12 subjects. The continuous EEG recordings were segmented into non-overlapping 1-s windows and represented as 1 × 30 × 128, corresponding to 30 channels and 128 temporal samples. In contrast to Dataset I, Dataset II provides a shorter temporal context and uses stage-based alert/fatigue labels, thereby allowing the proposed framework to be evaluated under a different segmentation and labeling setting.

Temporal segmentation and recombination (TSR) was used as a training-only augmentation strategy for both datasets. After the data partitions were established, each training sample was divided into eight temporal slices, and slices from samples belonging to the same class were recombined to generate additional training examples, resulting in an approximately fivefold increase in the corresponding training set. The same TSR configuration was used throughout the comparison experiments. For all evaluated models, TSR was restricted to source-domain training data and the labeled target-subject adaptation subset, whereas source-domain validation and held-out target test samples were not augmented.

### 3.2 Experimental setting

A cross-subject transfer protocol with labeled target-subject adaptation was adopted for model evaluation. For each target subject, all remaining participants constituted the source domain. The target-subject samples were stratified using a partition ratio of 0.5 into a labeled adaptation subset and a held-out test subset. The target split was generated once for each subject and remained fixed throughout the source-domain cross-validation procedure.

For the source domain, stratified 10-fold outer cross-validation was used. Within each outer fold, three-fold inner validation was performed for source-domain model selection. The selected source model was subsequently fine-tuned using only the labeled target-subject adaptation subset and evaluated on the corresponding held-out target test subset. The held-out target test data were not used for source training, fine-tuning, validation, early stopping, or model selection.

All models were implemented in PyTorch and optimized using Adam. The initial learning rate was set to 1 × 10^−3^ for Dataset I and 5 × 10^−4^ for Dataset II. During target-subject fine-tuning, the learning rate was reduced to 1 × 10^−4^ and 5 × 10^−5^ for Dataset I and Dataset II, respectively. Source-domain training was performed for a maximum of 200 epochs, with early stopping using a patience of 20 epochs based on source-domain validation performance. Target-subject fine-tuning was limited to 50 epochs. The batch size was fixed at 1,024.

For MSANet, component-specific dropout rates were set to 0.5 for the classifier, 0.2 for TRC, and 0.1 for SGNE. Explicit L1 and L2 regularization terms with coefficients of 1 × 10^−4^ and 1 × 10^−2^, respectively, were included in the training objective. The random seed was fixed at 2023 to improve the reproducibility of the experiments.

To evaluate the contribution of the proposed components, three ablated variants were constructed by removing MGPTG, SGNE, and ACG from the complete MSANet, respectively. All ablated variants were trained and evaluated using the same data partitions, optimization procedure, and cross-subject transfer protocol as the complete model.

### 3.3 Compared methods

The compared methods included EEGNet (Lawhern et al., 2018), BPR-STNet (Lin et al., 2024), ICNN (Cui et al., 2023), NHGNet (Xu et al., 2025), LGGNet (Ding et al., 2024), and the recent DCAMNet (Zhou et al., 2026). These methods cover compact convolutional, cross-subject, spatiotemporal, graph-based, and adaptive EEG representation learning approaches. All compared methods were evaluated under the same documented cross-subject transfer protocol and evaluation procedure.

### 3.4 Evaluation metrics

Accuracy (ACC), precision (PRE), sensitivity (SEN), specificity (SPE), and F1-score (F1) were used as the evaluation metrics, with fatigue treated as the positive class. Let *TP*, *TN*, *FP*, and *FN* denote the numbers of true positives, true negatives, false positives, and false negatives, respectively. The metrics are defined as


$$\begin{array}{l} \begin{array}{cccccc} ACC & = & \frac{TP+TN}{TP+TN+FP+FN}\;\\ PRE & = & \frac{TP}{TP+FP}\; & SEN & = & \frac{TP}{TP+FN}\\ SPE & = & \frac{TN}{TN+FP}\; & F1 & = & \frac{2\text{PRESEN}}{\text{PRE}+\text{SEN}} \end{array} \end{array}$$ (9)

SEN quantifies the proportion of fatigue samples correctly identified, whereas SPE measures the proportion of alert samples correctly classified as alert. PRE reflects the reliability of fatigue predictions, while F1 summarizes the balance between PRE and SEN. Because ACC alone may not fully characterize class-specific recognition behavior, these complementary metrics were jointly reported. This multi-metric evaluation strategy is consistent with recent EEG-based driver-fatigue studies that emphasize complementary performance assessment rather than reliance on a single summary measure (Chen et al., 2026c; Zhou et al., 2026).

### 3.5 Performance reporting and statistical analysis

For each target subject, performance was evaluated across the ten source-domain outer folds using the same held-out target test subset. The resulting metrics were first averaged across the ten source-domain outer-fold evaluations to obtain one subject-level value. Thus, the repeated evaluations reflect variation in source-domain cross-validation rather than repeated repartitioning of the target subject. The subject, rather than an individual source-domain fold, was therefore treated as the independent statistical unit, resulting in 11 subject-level observations for Dataset I and 12 for Dataset II. The values reported in Table 1 were then obtained by averaging these subject-level metrics across the 11 subjects in Dataset I and the 12 subjects in Dataset II.

**Table 1:** Performance comparison on Dataset I and Dataset II.

Dataset	Model	ACC (%)	PRE (%)	SEN (%)	SPE (%)	F1 (%)
Dataset I	EEGNet (Lawhern et al., 2018)	81.21	81.22	82.85	78.49	82.06
	BPR-STNet (Lin et al., 2024)	74.59	82.90	58.57	81.77	64.80
	ICNN (Cui et al., 2023)	82.33	82.84	82.75	81.91	82.58
	NHGNet (Xu et al., 2025)	81.63	80.39	83.89	77.28	82.63
	LGGNet (Ding et al., 2024)	74.98	75.05	77.16	72.69	75.24
	DCAMNet (Zhou et al., 2026)	79.41	81.05	79.91	78.82	79.78
	**MSANet (Ours)**	**83.64**	**83.72**	**84.78**	**82.41**	**83.74**
Dataset II	EEGNet (Lawhern et al., 2018)	66.65	67.40	54.53	78.17	57.86
	BPR-STNet (Lin et al., 2024)	59.17	57.62	73.69	54.66	67.51
	ICNN (Cui et al., 2023)	61.07	61.27	57.41	64.73	59.09
	NHGNet (Xu et al., 2025)	73.85	76.50	70.61	77.09	72.00
	LGGNet (Ding et al., 2024)	74.53	76.83	73.87	72.19	70.36
	DCAMNet (Zhou et al., 2026)	74.57	77.22	68.42	74.72	71.96
	**MSANet (Ours)**	**74.64**	**77.50**	**74.05**	**78.23**	**72.92**

All metrics are reported as percentages, and the highest numerical value in each column for each dataset is shown in bold.

ACC was treated as the primary statistical endpoint. For each dataset, overall differences among the seven evaluated models were first assessed using the Friedman test. MSANet was then compared with each baseline using two-sided paired Wilcoxon signed-rank tests. The six pairwise *p*-values within each dataset were adjusted using the Holm procedure to control for multiple comparisons. Statistical significance was defined as an adjusted *p*-value < 0.05.

## 4 Results

### 4.1 Comparison with existing methods

The performance comparison with six baseline models is presented in Table 1. The numerical results show stronger performance on Dataset I and a more competitive pattern on Dataset II; statistical inference was therefore used to distinguish descriptive differences from improvements supported at the subject level.

On Dataset I, MSANet achieved 83.64% ACC, 83.72% PRE, 84.78% SEN, 82.41% SPE, and 83.74% F1. Compared with the strongest metric-specific baselines, the corresponding improvements were 1.31, 0.82, 0.89, 0.50, and 1.11 percentage points, respectively.

On Dataset II, MSANet obtained 74.64% ACC, 77.50% PRE, 74.05% SEN, 78.23% SPE, and 72.92% F1. The strongest competing values were 74.57% ACC and 77.22% PRE from DCAMNet, 73.87% SEN from LGGNet, 78.17% SPE from EEGNet, and 72.00% F1 from NHGNet. The corresponding margins were 0.07, 0.28, 0.18, 0.06, and 0.92 percentage points. These results indicate competitive rather than substantial gains on Dataset II.

The Friedman test indicated significant overall differences among the seven models on both Dataset I (*p* = 0.0022) and Dataset II (*p* < 0.001). After Holm correction, significant pairwise differences were observed between MSANet and BPR-STNet (*p*_adj_ = 0.0117) and between MSANet and LGGNet (*p*_adj_ = 0.0342) on Dataset I. On Dataset II, significant pairwise differences were observed between MSANet and BPR-STNet (*p*_adj_ = 0.0029) and between MSANet and ICNN (*p*_adj_ = 0.0049). The remaining pairwise comparisons did not reach statistical significance after Holm correction. These findings indicate that numerical differences in mean performance do not necessarily correspond to statistically reliable subject-level differences, particularly when performance varies across individuals.

The baseline results also reveal considerable variation across evaluation metrics. For example, BPR-STNet showed relatively high PRE but substantially lower SEN on Dataset I, whereas the opposite trend was observed on Dataset II. This supports the joint use of ACC, PRE, SEN, SPE, and F1 when evaluating fatigue-recognition models.

### 4.2 Ablation study

The module-removal results are presented in Table 2.

**Table 2:** Ablation results on Dataset I and Dataset II.

Dataset	Variant	ACC (%)	PRE (%)	SEN (%)	SPE (%)	F1 (%)
Dataset I	MSANet w/o MGPTG	81.29	80.79	84.02	78.47	81.93
	MSANet w/o SGNE	80.40	80.16	82.65	78.07	80.90
	MSANet w/o ACG	80.30	80.01	83.58	76.93	81.19
	MSANet	**83.64**	**83.72**	**84.78**	**82.41**	**83.74**
Dataset II	MSANet w/o MGPTG	72.65	75.03	69.29	76.01	70.53
	MSANet w/o SGNE	73.78	76.66	70.98	76.58	72.30
	MSANet w/o ACG	72.24	75.49	69.46	75.02	70.59
	MSANet	**74.64**	**77.50**	**74.05**	**78.23**	**72.92**

All metrics are reported as percentages, and the highest numerical value in each column for each dataset is shown in bold.

On Dataset I, removing ACG produced the largest decreases in ACC, PRE, and SPE, whereas removing SGNE resulted in the largest reductions in SEN and F1. Removing MGPTG also reduced all five metrics.

On Dataset II, removing MGPTG caused the largest decreases in PRE, SEN, and F1, whereas removing ACG produced the largest losses in ACC and SPE. Removing SGNE also reduced all five metrics. Overall, removing any of the three modules reduced performance, although the magnitude of the reduction varied across the two datasets.

## 5 Discussion

The results indicate that MSANet achieves competitive fatigue-recognition performance under cross-subject transfer across two EEG datasets with different temporal-window settings and labeling protocols. The mean performance is stronger on Dataset I, whereas the numerical margins over several strong baselines on Dataset II are comparatively small. The subject-level statistical analysis further showed that significant differences were limited to selected baseline comparisons after Holm correction. Accordingly, the comparative results are interpreted as competitive performance with selected statistically supported differences rather than uniform superiority over all competing methods.

Compared with existing convolutional and graph-based approaches, the main distinction of MSANet lies in the introduction of dedicated mechanisms at different stages of the temporal–graph pipeline rather than in dynamic graph learning itself. LGGNet and NHGNet mainly emphasize spatial or dynamic graph representation learning, whereas DCAMNet focuses on adaptive feature extraction across spectral, spatial, and temporal dimensions. In contrast, MSANet additionally introduces MGPTG for locally continuous temporal modeling before compression, SGNE for explicit node enhancement before dynamic adjacency estimation, and ACG for gated dual-geometry classification after graph convolution. These mechanisms address different modeling operations and extend the temporal–graph pipeline beyond the dynamic graph formulation alone.

The ablation results suggest complementary roles for the three proposed components. The performance reduction associated with removing MGPTG was more pronounced on Dataset II, which uses shorter 1-s input segments. Removing SGNE also reduced performance, consistent with its intended role in enhancing node representations before graph construction. Similarly, the ACG ablation is consistent with a beneficial role for combining cosine-based and affine classification geometries at the decision stage. These observations describe the functional patterns associated with the proposed modules and should not be interpreted as evidence of specific physiological mechanisms.

Several limitations should be considered. First, labeled target-subject data were used for adaptation before evaluation on a held-out subset from the same target subject. Therefore, the present protocol evaluates cross-subject transfer with target-subject adaptation rather than strict subject-independent generalization to a completely unseen subject. Second, both datasets were collected in simulated-driving environments and were limited to binary alert/fatigue discrimination, which may restrict generalization to more complex real-world driving states. Third, the variability of the subject-level results and the limited statistical separation from several stronger baselines indicate the need for validation on larger and more heterogeneous datasets. In addition, the learned adjacency matrices represent model-derived channel relations and should not be directly interpreted as physiological functional connectivity. From an aging-related perspective, reliable fatigue monitoring may be particularly relevant to older drivers because age-associated cognitive and behavioral changes can affect driving safety. However, the current datasets do not provide an age-stratified older-driver cohort. Accordingly, the present findings should not be interpreted as evidence of age-specific effectiveness, and validation in dedicated older-adult driving cohorts is required before extending the conclusions across age groups.

## 6 Conclusion

In this work, MSANet was developed for cross-subject transfer in EEG-based driver fatigue recognition. The framework integrates MGPTG for locally continuous temporal modeling, SGNE for node enhancement before dynamic graph construction, and ACG for gated dual-geometry classification, respectively. Across two public EEG datasets, MSANet showed strong numerical performance on Dataset I and competitive performance on Dataset II. Subject-level statistical analysis identified significant differences for selected baseline comparisons after Holm correction, while the remaining comparisons did not reach statistical significance.

These findings are consistent with the proposed stage-specific modeling framework under the evaluated transfer setting, while also showing that superiority is not uniform across all comparison methods. Future work will focus on stricter subject-independent evaluation, real-driving scenarios, and validation on larger and more heterogeneous datasets, including dedicated older-adult driving cohorts, to examine the robustness of the framework across age groups.

## Data Availability

Publicly available datasets were analyzed in this study. Dataset I is available in the Figshare repository under the title “Multi-channel EEG recordings during a sustained-attention driving task (pre-processed dataset)” at https://doi.org/10.6084/m9.figshare.7666055.v3. Dataset II is available in the Figshare repository under the title “The original EEG data for driver fatigue detection” at https://doi.org/10.6084/m9.figshare.5202739.
